# Jiangshui-derived *Lactobacillus plantarum* GR-4 enhances the therapeutic efficacy of PD-1/PD-L1 inhibitors against gastric cancer

**DOI:** 10.3389/fimmu.2026.1987320

**Published:** 2026-10-29

**Authors:** Na Wang, Kai Wang, Huawen Han, Jingwen Fan, Feixue Song, Hao Chen, Xiangkai Li

**Affiliations:** 1 Department of Oncology, The Second Hospital & Clinical Medical School, Lanzhou University, Lanzhou, Gansu, China; 2 Departments of Orthopedics, The Second Hospital & Clinical Medical School, Lanzhou University, Lanzhou, Gansu, China; 3 Centre for Grassland Microbiome, State Key Laboratory of Herbage Improvement and Grassland Agro-ecosystems, College of Pastoral Agricultural Science and Technology, Lanzhou University, Lanzhou, Gansu, China; 4 Ministry of Education Key Laboratory of Cell Activities and Stress Adaptations, School of Life Sciences, Lanzhou University, Lanzhou, Gansu, China; 5 The Second Hospital & Clinical Medical School, Lanzhou University, Lanzhou, Gansu, China

**Keywords:** gastric cancer, immune microenvironment, Jiangshui, Lactobacillus plantarum, PD-1 inhibitor

## Abstract

**Objective:**

To investigate whether the Jiangshui-derived probiotic *Lactobacillus plantarum* GR-4 can enhance gastric cancer sensitivity to PD-1 inhibitors and reshape the tumor immune microenvironment.

**Methods:**

A mouse gastric cancer model was established. Mice received oral GR-4 monotherapy, PD-1 blocking antibody monotherapy, or combined GR-4 and PD-1 antibody treatment. *In vivo* antitumor efficacy was evaluated by measuring tumor weight. We assessed CD8^+^ T-cell infiltration, cytokines related to T-cell activation and differentiation, and the expression of the activation marker CD69 on CD3^+^/CD8^+^ T cells within tumor tissues. 16S rRNA sequencing of gut microbiota and fecal metabolomic analysis were performed to explore the regulatory effects of GR-4 on gut microbiota composition and metabolic profiles. *In vitro*, human gastric cancer BGC-823 cells were treated with GR-4 combined with PD-1 intervention, and transcriptome sequencing was applied to further validate the underlying molecular regulatory mechanisms.

**Results:**

*In vivo* results demonstrated that combined GR-4 and PD-1 antibody treatment exerted prominent synergistic antitumor activity, effectively suppressing tumor growth while markedly mitigating PD-1-associated body weight loss (p < 0.01). Further assays revealed that GR-4 synergistically elevated tumor-infiltrating CD3^+^/CD8^+^ T-cell levels and upregulated CD69 expression on these cells, thereby remodelling the tumor immune microenvironment. Additionally, GR-4, whether administered alone or combined with PD-1 antibody, optimized gut microbiota structure, increased the abundance of probiotic genera including *Ligilactobacillus* and *Bacteroides*, reversed intestinal metabolic disturbances, and raised the levels of short-chain fatty-acid-related metabolites such as ethoxyacetic acid and 2,5-dihydroxybenzoic acid. Transcriptomic sequencing showed significant enrichment of the PD-1 checkpoint pathway in the GR-4 group versus Ctrl (p=0.01), whereas no significant enrichment was observed between the PD-1 inhibitor group and GR-4/PD-1 inhibitor group (p=0.14).

**Conclusion:**

This study is the first to confirm that *Lactobacillus plantarum* GR-4 synergizes with PD-1 inhibitors to restrain gastric cancer progression via T-cell activation and elevated intratumoral T-cell infiltration. These findings provide novel theoretical evidence and a potential combinatorial immunotherapeutic strategy for gastric cancer.

## 1 Introduction

Gastric cancer is a highly prevalent malignancy worldwide and poses a substantial public-health burden in China, where its incidence and mortality consistently rank among the highest of all cancers (1). Conventional therapeutic modalities yield limited clinical benefits, and advanced gastric cancer still relies heavily on chemotherapy, whose therapeutic efficacy has reached a plateau. Immune checkpoint inhibitors (ICIs), especially antibodies targeting programmed death-1 (PD-1), have become an indispensable therapeutic option for advanced gastric cancer (2, 3). Nevertheless, widespread primary or acquired resistance leads to unsatisfactory response rates for monotherapy, with objective response rates generally ranging from 10% to 26% (4). This clinical bottleneck has driven researchers to actively develop combinatorial therapeutic strategies that overcome resistance by remodelling the immunosuppressive tumor microenvironment (TME) (5).

For instance, several studies have reported that combining anti-angiogenic agents (e.g., apatinib (rivoceranib) or anlotinib) with PD-1 inhibitors can synergistically amplify the antitumor activity of PD-1 blockade (6). However, other investigations have noted suboptimal efficacy for certain combination regimens, which may even increase adverse event risks (7). Accordingly, identifying novel combinatorial strategies that specifically elicit antitumor immunity while maintaining a favourable safety profile is critically needed.

Against this backdrop, probiotics have been demonstrated to exert antitumor effects by modulating host immunity, and restoring gut microbiota homeostasis represents a key approach to optimize cancer immunotherapy. Probiotics indirectly suppress gastric carcinogenesis by sustaining microbiota homeostasis and regulating metabolic processes (8). First, probiotics rectify gastric dysbiosis induced by *Helicobacter pylori* infection, reduce colonization by pathogenic bacteria such as *Fusobacterium nucleatum*, and restore commensal microbiota diversity. Second, probiotic metabolites (e.g., butyrate) repress tumor cell proliferation by activating the AMPK pathway and upregulating DNA damage-inducible transcript 3 (DDIT3), triggering cell-cycle arrest and apoptosis. For example, ferrichrome, a siderophore protein produced by *Lactobacillus casei*, inhibits DNA synthesis and induces apoptosis in oesophageal cancer models with low toxicity toward normal cells (9).

Furthermore, probiotics strengthen intestinal barrier integrity, attenuate endotoxin translocation, and mitigate systemic-inflammation-driven gastric cancer progression. Animal studies have shown that probiotic intervention substantially delays tumor growth in gastric-cancer-bearing mice. When combined with chemotherapy, probiotics can alleviate chemotherapy-related adverse effects such as 5-fluorouracil (5-FU)-induced myelosuppression (10).

In recent years, Jiangshui-derived probiotics have attracted growing research interest. Jiangshui is a traditional fermented food originating from Northwest China. Produced via microbial fermentation, it is rich in probiotics, vitamin C, organic acids and other nutrients, and exerts benefits including promoting digestion, lowering cholesterol, and supporting intestinal health (11). Lactic acid bacteria play central roles during Jiangshui fermentation: they ferment carbohydrates to generate lactic acid, suppress pathogenic bacterial overgrowth, preserve mucosal-barrier integrity, and boost host immune competence (12). Moreover, antimicrobial metabolites (e.g., bacteriocins) secreted by lactic acid bacteria carry low resistance-inducing potential. They are promising candidates for clinical anti-infective therapy and also display encouraging activity against colorectal tumor growth (13).

Our prior work demonstrated that *Limosilactobacillus fermentum* GR-3, a probiotic strain isolated from Jiangshui, markedly inhibits colorectal tumor development. Mice receiving GR-3 intervention exhibited fewer colon tumors and ameliorated intestinal manifestations such as diarrhoea and rectal bleeding (14, 15). Further follow-up studies identified another Jiangshui-isolated strain, *Lactobacillus plantarum* GR-4. This strain reduces conjugated bile-acid concentrations and effectively mitigates gastroenteritis; it exhibits superior antitumor potency relative to GR-3, particularly against gastric cancer cell proliferation. Nevertheless, the exact molecular mechanisms underlying GR-4-mediated anti-gastric-cancer activity remain uncharacterized. In particular, whether and how GR-4 can synergistically augment the efficacy of current gastric cancer immunotherapies (e.g., PD-1/PD-L1 inhibitors) is still unclear.

The present study systematically assesses the synergistic effects of *Lactobacillus plantarum* GR-4 combined with PD-1 antibody on gastric cancer cell killing and tumor-immune-microenvironment remodelling using *in vivo* and *in vitro* experiments. Based on transcriptomic profiling, we preliminarily decipher its potential molecular mechanisms. This work provides the first systematic evidence that GR-4 synergizes with PD-1 antibody to constrain gastric cancer growth by activating T-cell signalling cascades, promoting tumor-cell apoptosis, and improving immune-cell infiltration within the tumor microenvironment.This multi-dimensional analytical framework helps reveal the immunomodulatory potential of GR-4 and supports the translational value of microbiota-targeted adjuvant immunotherapy for gastric cancer (16).

## 2 Materials and methods

### 2.1 Cell culture and probiotic treatment

*Lactobacillus plantarum GR-4* was isolated from Jiangshui, a traditional fermented food from the Northwest Plateau of China. Jiangshui was inoculated into MRS medium at 3% (v/v) and incubated anaerobically at 37 °C for 24 h. Single colonies were picked, and genomic DNA was extracted using a commercial kit. GR-4 formed milky-white, convex colonies (2–3 mm) with regular margins and moist surfaces on solid medium. 16S rRNA sequencing confirmed 98% sequence homology with *L. plantarum JCM 1149*, and phylogenetic analysis verified its taxonomic classification as *L. plantarum GR-4* (17)*. L. plantarum* GR-4 (isolated and identified from Lanzhou Jiangshui by the Key Laboratory of Cell Activities and Stress Adaptations, Ministry of Education, School of Life Sciences, Lanzhou University) was cultured anaerobically in MRS medium (Oxoid, UK) at 37 °C for 24 h. Bacterial pellets were harvested, washed with PBS, and resuspended to the required working concentration (OD_600_ = 0.6 corresponds to approximately 1×10^9^ CFU/mL). Human gastric cancer BGC-823 cells (a gift from the Gansu Provincial Key Laboratory of Gastroenterology, Lanzhou University), normal human gastric mucosal epithelial GES-1 cells (a gift from the Gansu Provincial Key Laboratory of Digestive System Tumors, Lanzhou University Second Hospital), and mouse forestomach carcinoma MFC cells (purchased from Shanghai Jinyuan Biotechnology Co., Ltd.) were cultured in RPMI-1640 medium (HyClone, USA) supplemented with 10% fetal bovine serum (FBS, Gibco, USA), 100 U/mL penicillin, and 100 μg/mL streptomycin, in a 5% CO_2_ incubator maintained at 37 °C.

### 2.2 Splenic T cell isolation and co-culture experiments

Spleens were aseptically harvested from 6-8-week-old male C57 mice (purchased from Beijing Vital River Laboratory Animal Technology Co., Ltd.). Single-cell suspensions were generated by grinding tissues through a 70 μm cell strainer. CD3^+^ T cells were enriched using a mouse T-cell enrichment column (Miltenyi Biotec, Germany), achieving >95% purity. Gastric cancer cells (mouse-derived MFC) were pre-labelled with 5 μM CFSE and seeded into 96-well plates at 1×10^5^ cells per well.

Co-culture experimental groups were set as follows: (1) Blank group (CFSE-labelled gastric cancer cells only); (2) Blank+PD-1 group (gastric cancer cells + 10 μg/mL anti-PD-1 antibody, BioLegend, clone RMP1-14); (3) Blank+GR-4 group (gastric cancer cells + 1×10^7^ CFU/mL GR-4; this concentration was validated in preliminary assays to exert no significant cytotoxicity against gastric cancer cells); (4) Blank+PD-1+GR-4 group (gastric cancer cells + PD-1 antibody + GR-4); (5) Co-culture group (MFC cells + 1×10^6^ splenic T cells; effector-to-target ratio = 10:1); (6) Co-culture+PD-1 group (co-culture system plus PD-1 antibody); (7) Co-culture+GR-4 group (co-culture system plus GR-4); (8) Co-culture+GR-4+PD-1 group (co-culture system plus PD-1 antibody and GR-4). For cell experiments with GR-4 intervention, GR-4 at the logarithmic growth phase (OD = 0.3) was used uniformly. After harvesting the bacteria, they were washed twice with PBS and then resuspended in PBS to an OD of 0.6. Briefly, 10 μL of GR-4 bacterial suspension was added into 5 mL complete culture system, and the cells were incubated for 3 hours. After that, the medium containing GR-4 was discarded, and cells were washed twice with PBS. Fresh complete medium was then replaced for subsequent culture, or PD-1 inhibitor treatment was applied for another 24 h.

All groups were incubated at 37 °C for 48 h. After incubation, cells were collected, and 125 μL cell suspension was transferred into flow cytometry tubes. Negative-control tubes (no antibodies), single-stain compensation tubes (single antibody only), and sample tubes were prepared. Sample tubes received 5 μL PE-conjugated anti-mouse CD3ϵ, 5 μL PerCP-Cy5.5-conjugated anti-mouse CD4, 5 μL FITC-conjugated anti-mouse CD8, and 5 μL APC-conjugated anti-mouse CD69. Tubes were vortex-mixed and incubated in the dark at room temperature for 15 min. Each group contained n = 3 biological replicates, and the whole experiment was repeated three times.

### 2.3 *In vivo* mouse tumor model experiments

615-strain male mice (5-6 weeks old; body weight 18 ± 2 g) were purchased from the Experimental Animal Center of the Institute of Hematology, Chinese Academy of Medical Sciences (License No.: SCXK(Jin)20250001). Each mouse received a subcutaneous inoculation of 0.1 mL MFC cell suspension (1×10^7^ cells/mL) into the right flank. Mice were randomly assigned to four groups: Group A (control group): 0.1 mL PBS administered by gavage daily, six times per week; Group B (GR-4 group): 0.1 mL GR-4 bacterial suspension administered by gavage daily, six times per week; Group C (PD-1 group): 100 μg PD-1 antibody per mouse via intraperitoneal injection once weekly; Group D (PD-1+GR-4 group): intraperitoneal PD-1 antibody combined with intragastric GR-4 bacterial suspension.

Starting on the day of tumour-cell inoculation, mouse body weight was recorded every three days. From day 7 post-inoculation, tumour length and width were measured using callipers, and tumour volume was calculated using the formula: Tumor volume = 0.52 × length × width². On day 15 post-inoculation, mouse serum and faecal samples were collected and snap-frozen in liquid nitrogen for downstream analyses.

### 2.4 Serum cytokine detection

Serum concentrations of IL-2, IL-4, IL-10, IFN-γ and TGF-β were quantified using commercial ELISA kits (R&D Systems, USA), strictly following the manufacturer’s protocols. Each sample was assayed in triplicate. Optical-density values were read at 450 nm on a Bio-Rad microplate reader, and cytokine concentrations (pg/mL) were calculated against standard curves.

### 2.5 Immunofluorescence staining

Tumour tissues were fixed in 4% paraformaldehyde, paraffin-embedded, and sectioned to 5 μm thickness. Following deparaffinization and rehydration, antigen retrieval was performed in pH 6.0 citrate buffer at 95 °C for 20 min, followed by blocking with 5% goat serum for 30 min. Sections were incubated overnight at 4 °C with primary antibodies: rabbit anti-mouse CD3 (1:200, Abcam, ab16669) and rat anti-mouse CD4 (1:200, BD Pharmingen) or rat anti-mouse CD8 (1:200, BD Pharmingen). After PBS washing, secondary antibodies were applied: Alexa Fluor 488-conjugated goat anti-rabbit IgG (1:1000, Invitrogen) and Alexa Fluor 594-conjugated goat anti-rat IgG (1:1000, Invitrogen), followed by 1-h incubation at room temperature in the dark. Nuclei were counterstained with 1 μg/mL DAPI (Sigma). Five random 200× magnification fields were captured under a confocal microscope. CD3^+^CD4^+^ and CD3^+^CD8^+^ double-positive cells were enumerated using ImageJ software, and positive-cell counts per mm² tissue area were computed (18, 19).

### 2.6 Transcriptome sequencing

BGC-823 cells were harvested following treatment with GR-4 and/or PD-1 blockade, and total RNA was extracted using TRIzol reagent (Invitrogen). RNA purity, concentration and integrity (RIN ≥ 8) were assessed using a Nanodrop 2000 spectrophotometer (Thermo) and an Agilent 2100 Bioanalyzer (Agilent, USA). RNA libraries were constructed with the Illumina TruSeq RNA Library Prep Kit v2, and paired-end 150-bp sequencing was performed on the Illumina NovaSeq 6000 platform. Raw sequencing reads were quality-controlled with FastQC, and low-quality reads were discarded. Clean reads were mapped to the human reference genome GRCh38.p14 (release-113) using TopHat2 (v2.1.1), and gene expression levels (FPKM) were calculated with Cufflinks (v2.2.1). Differentially expressed genes (DEGs) were identified via DESeq2 with cut-offs |Fold Change| ≥ 2 and FDR ≤ 0.05. KEGG database resources were used for pathway-enrichment analysis.

### 2.7 Metabolomics detection

Liquid-nitrogen-preserved mouse faecal samples were submitted to Beijing Qingke Biotechnology Co., Ltd. for sample pre-processing and liquid chromatography-tandem mass-spectrometry detection. Raw datasets underwent pre-processing and quality-control filtering. Data analysis was conducted on the online analytical platform provided by Qingke Biotechnology.

### 2.8 16S rRNA gene sequencing and bioinformatics analysis

Frozen faecal samples were sent to Beijing Qingke Biotechnology Co., Ltd. The V3-V4 hypervariable region of the 16S rRNA gene was amplified using primers 338F (5′-actcctacgggaggcagcag-3′) and 806R (5′-ggactachvgggtwtctaat-3′). Subsequent bioinformatic analyses were completed using the company’s standard analytical pipeline.

### 2.9 Statistical analysis

All data are presented as mean ± standard deviation (Mean ± SD). Statistical computations were performed in GraphPad Prism 9.0 (GraphPad Software, USA). Two-group comparisons were conducted using independent-samples t-tests. For multi-group comparisons, one-way or two-way analysis of variance (ANOVA) was applied, followed by Tukey’s or Dunnett’s post-hoc multiple-comparison tests. A p-value < 0.05 was defined as statistically significant.

## 3 Results

### 3.1 GR-4 synergistically enhances the antitumor effect of PD-1 in a mouse model of gastric cancer

To characterize the antitumor potential of GR-4, subcutaneous MFC tumour xenografts were established in 615 mice (**Figure 1A**). Interventions were initiated seven days after tumour-cell inoculation, and tumour dimensions were measured every three days using callipers (**Figure 1B**). Systemic toxicity was monitored by serial body-weight recording. On day 19 relative to inoculation (**Figure 1C**), mice receiving PD-1 monotherapy exhibited ~10.8% body-weight loss relative to baseline. Moderate weight reduction was observed in the GR-4 group (\0.8%) and GR-4/PD-1 combination group (\1.8%), with markedly better weight maintenance compared with PD-1 monotherapy. The control group displayed ~5.4% weight loss driven by progressive tumour burden. At day 19, body weights in all other groups were significantly higher than those in the PD-1 monotherapy group (p < 0.01), indicating that GR-4 preserves body weight in tumour-bearing mice. Tumour-growth kinetic analyses (**Figure 1D**) revealed that the GR-4/PD-1 combination group achieved the strongest tumour-growth-suppressive effect relative to controls. Although GR-4 monotherapy slightly reduced tumour size, this difference did not reach statistical significance, demonstrating that oral GR-4 alone exerts minimal intrinsic antitumor activity. Nevertheless, GR-4 robustly synergized with PD-1 blocking antibody to amplify PD-1-mediated antitumor responses. Consistent findings were obtained for excised terminal tumour weights (**Figures 1E, F**).

**Figure 1:**
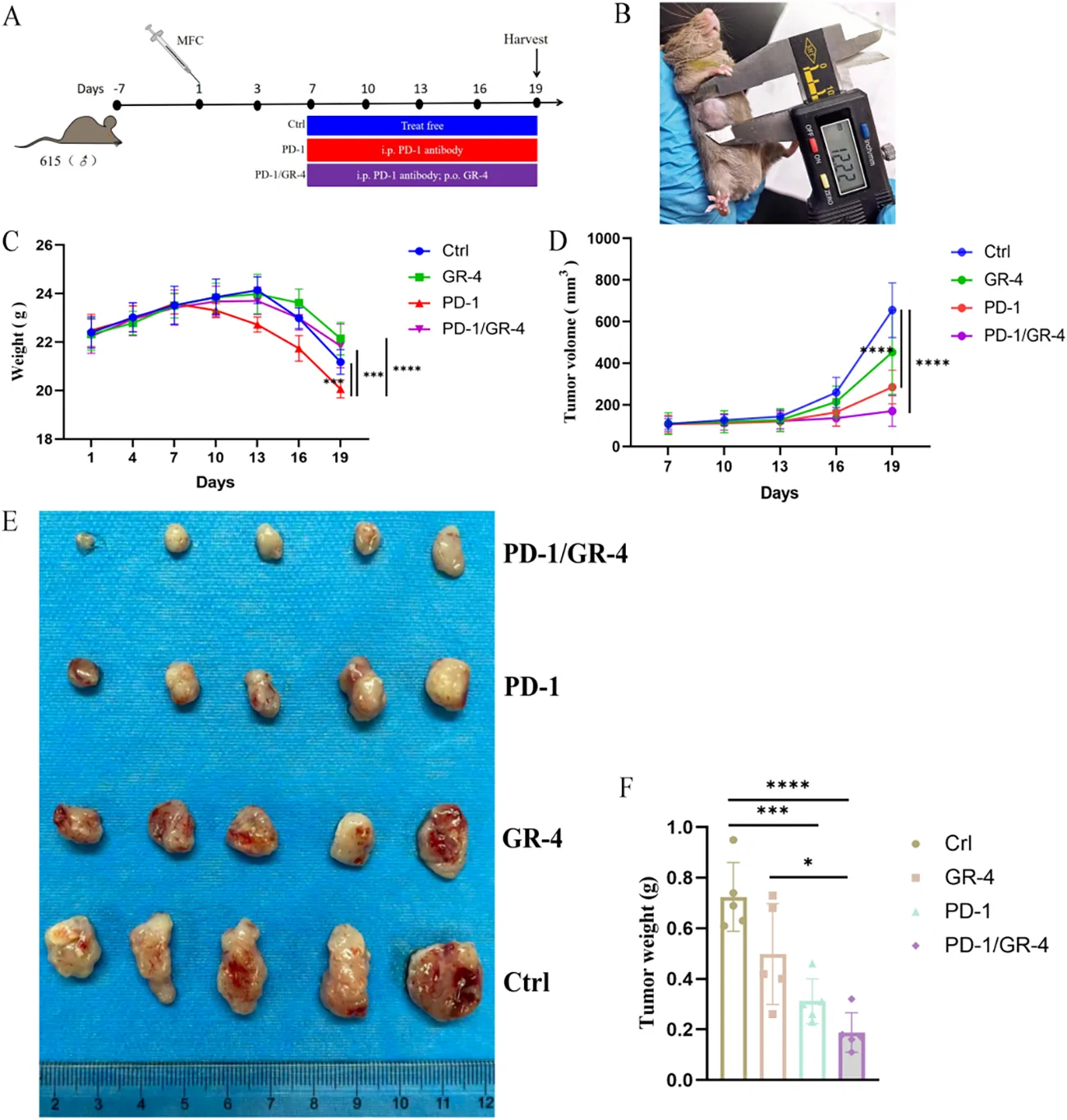
GR-4 synergistically inhibits tumor growth *in vivo* with PD-1 immunotherapy. **(A)** Schematic diagram of the experimental design. **(B)** Subcutaneous tumor measurement. **(C)** Body weight changes of tumor-bearing mice over time. **(D)** Tumor volume changes over time. **(E)** Excised tumor tissue masses. **(F)** Weight of excised tumor tissues. Data are presented as mean ± SD, n=5 per group, *p< 0.05, ***p< 0.001, ****p< 0.0001.

### 3.2 GR-4 synergizes with PD-1 to improve the tumor immune microenvironment and exert antitumor effects

To dissect the mechanisms whereby GR-4 augments PD-1-driven antitumor immunity, multiplex immunofluorescence staining was performed on excised tumour tissues to quantify CD4^+^ and CD8^+^ T-cell infiltration (**Figure 2A**). Compared with the control group, PD-1 monotherapy significantly increased intratumoral CD3^+^/CD8^+^ T-cell density. The most prominent elevation of CD3^+^/CD8^+^ T-cell tumour infiltration was detected in the GR-4 plus PD-1 intervention group. CD3^+^/CD4^+^ T-cell infiltration increased modestly but remained at low overall levels. These observations suggest that GR-4-driven potentiation of PD-1 antitumor efficacy is linked to remodelling of the tumour immune microenvironment. To further interrogate underlying mechanisms, we measured serum levels of IL-2, IL-4, IL-10, TGF-β and IFN-γ in peripheral blood (**Figures 2B–F**). Relative to controls, circulating IL-2 was significantly elevated in the GR-4/PD-1 combination group (p < 0.01, **Figure 2B**), whereas PD-1 monotherapy produced no significant IL-2 upregulation. IL-4 concentrations showed no inter-group differences (**Figure 2C**). IL-10 levels were significantly increased in the combination group (p < 0.01, **Figure 2D**). No significant differences in TGF-β were observed across groups (**Figure 2E**). IFN-γ levels were markedly elevated upon combined GR-4 and PD-1 treatment (p < 0.01, **Figure 2F**). Collectively, these data indicate that GR-4 boosts PD-1 therapeutic efficacy by modulating serum levels of cytokines associated with CD8^+^ T-cell activation and differentiation, thereby promoting intratumoral CD8^+^ T-cell accumulation.

**Figure 2:**
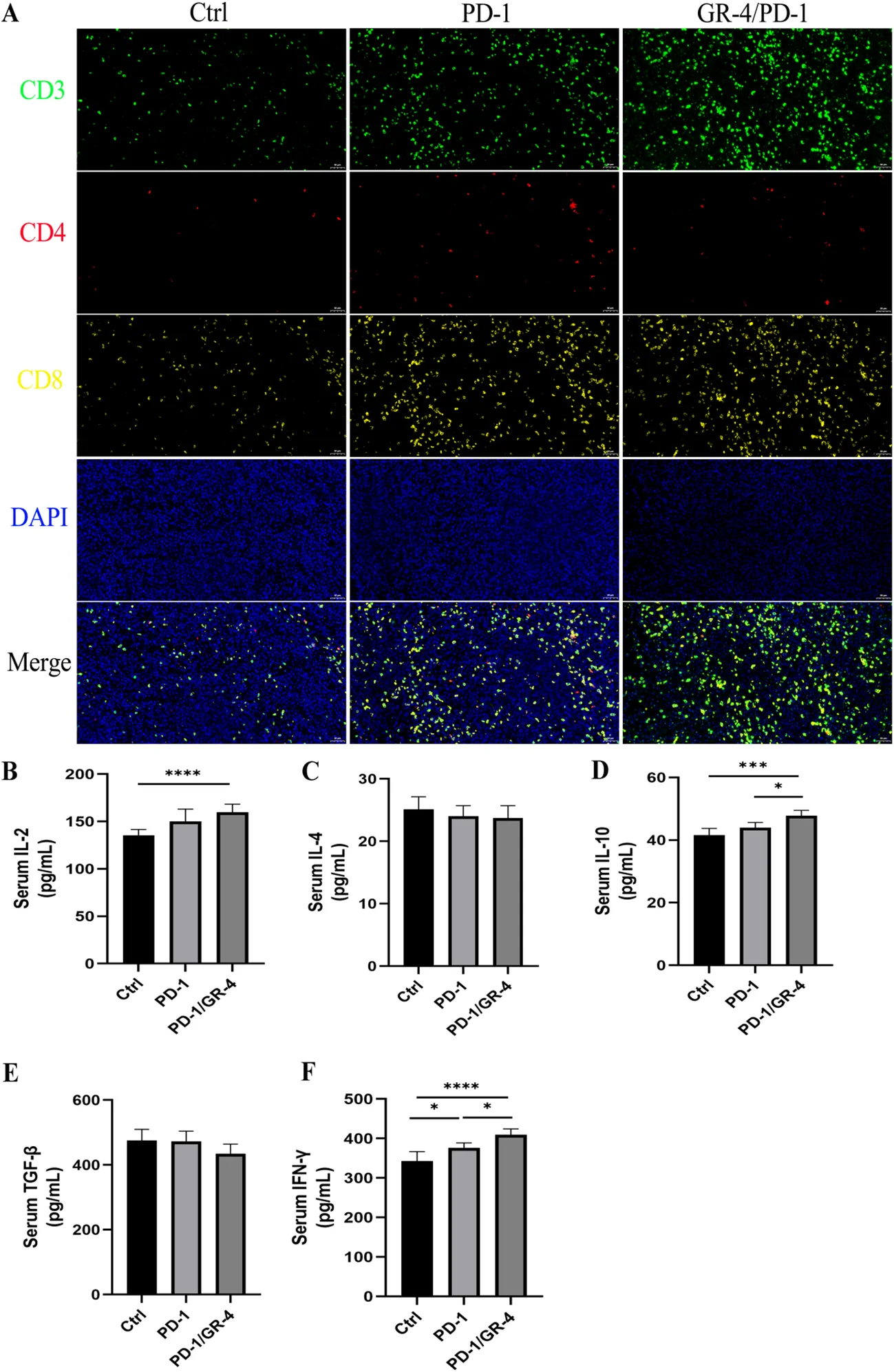
GR-4 synergistically regulates the immune microenvironment *in vivo* with PD-1 immunotherapy in tumor-bearing mice. **(A)** Immunofluorescence detection of tumor-infiltrating CD3+CD4+/CD8+ T cell levels, Green: CD3+; Red: CD4+; Yellow: CD8+; Blue: DAPI. **(B)** Mouse serum IL-2 levels. **(C)** Mouse serum IL-4 levels. **(D)** Mouse serum IL-10 levels. **(E)** Mouse serum TGF-β levels. **(F)** Mouse serum IFN-γ levels. Data are presented as mean ± SD, n=3 per group, *p< 0.05, ***p< 0.001, ****p< 0.0001.

### 3.3 GR-4 synergistically increases CD69 expression levels on CD3+/CD8+ T cells

To further explore how GR-4 combined with PD-1 shapes tumour-infiltrating T-cell phenotypes, we isolated splenic lymphocytes *in vitro* and treated cells with GR-4 and/or PD-1 blockade. Flow cytometry was used to quantify CD69 expression on CD3^+^/CD4^+^ and CD3^+^/CD8^+^ T-cell subsets. GR-4 and/or PD-1 intervention significantly down-regulated CD69 expression on CD3^+^/CD4^+^ T cells, with maximal reduction in the GR-4/PD-1 group (**Figures 3A, B**). By contrast, either GR-4 or PD-1 monotherapy significantly increased CD69 expression on CD3^+^/CD8^+^ T cells, and combination treatment yielded the most pronounced CD69 upregulation (**Figures 3C, D**). To recapitulate the *in vivo* tumour-microenvironment context, MFC tumour cells were co-cultured with mouse splenic lymphocytes in the presence or absence of GR-4 and PD-1 antibody, and CD69 expression was assessed. Under co-culture conditions, GR-4 and/or PD-1 treatment significantly decreased CD69 levels on CD3^+^/CD4^+^ T cells, with the greatest decrease observed in the GR-4/PD-1 group (**Figures 3E, F**). For CD3^+^/CD8^+^ T cells, single-agent GR-4 or PD-1 treatment increased CD69 expression, and dual treatment induced the strongest upregulation (**Figures 3G, H**). CD69 is a key marker during T-cell activation. Notably, GR-4 and PD-1 blockade exerted opposing effects on CD69 expression in CD4^+^ versus CD8^+^ T-cell populations. This implies that GR-4 and PD-1 may share partially overlapping, or even synergistic, mechanisms for reshaping the tumour immune microenvironment.

**Figure 3:**
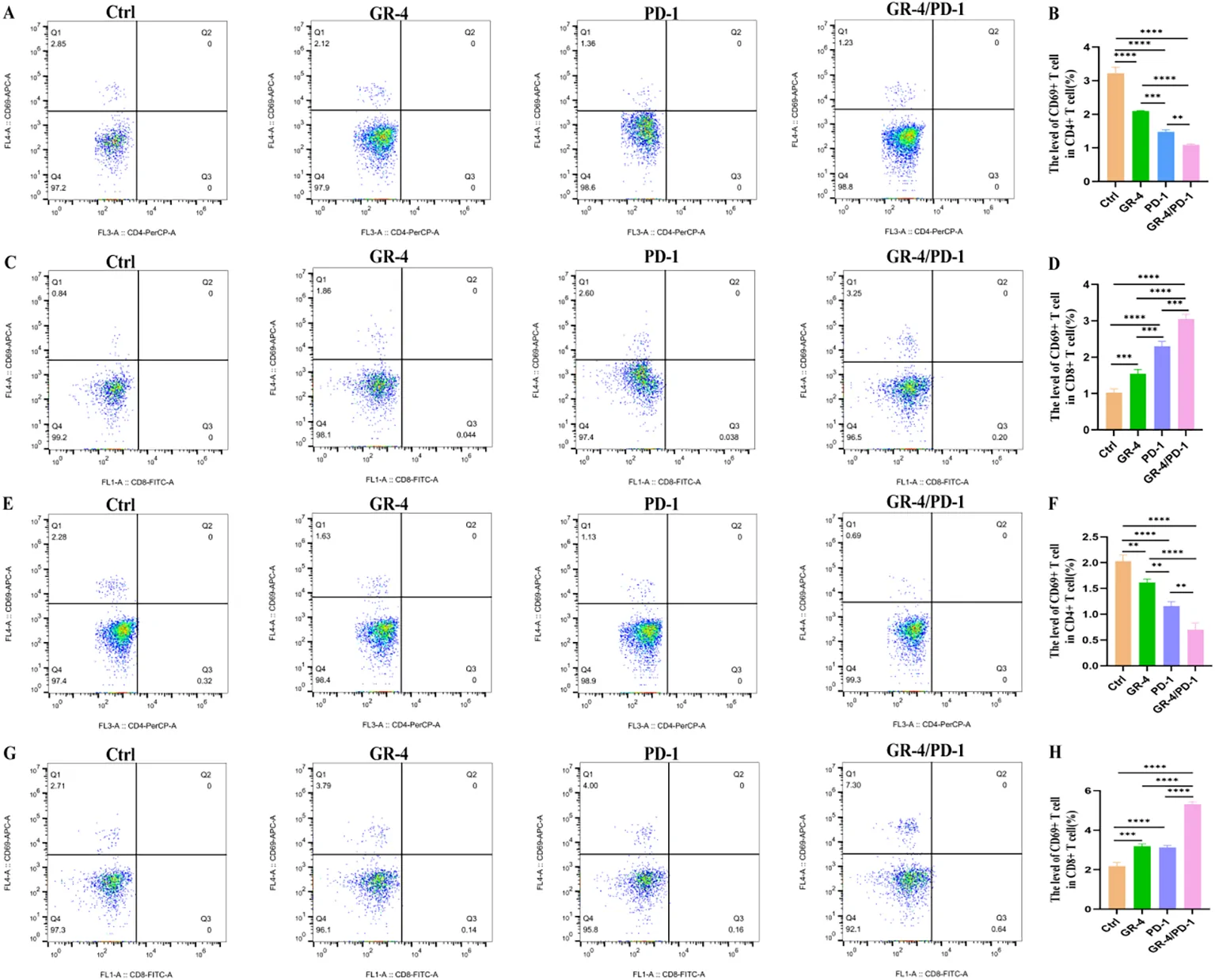
Effect of GR-4 synergizing with PD-1 blocker on CD69 expression in T cells. **(A, B)** Effect of GR-4 on CD69 expression in CD4+ T cells. **(C, D)** Effect of GR-4 on CD69 expression in CD8+ T cells. **(E, F)** Effect of GR-4 on CD69 expression in CD4+ T cells under co-culture with MFC cells. **(G, H)** Effect of GR-4 on CD69 expression in CD8+ T cells under co-culture with MFC cells. Data are presented as mean ± SD, n=3 per group **p< 0.01, ***p< 0.001, ****p< 0.0001.

### 3.4 GR-4 can exert antitumor effects by regulating the gut microbiota and metabolites to influence the body’s immune microenvironment

To dissect gut-microbiota-dependent pathways by which GR-4 regulates host immunity, we performed 16S rRNA sequencing on faecal samples (**Figure 4**). Alpha-diversity metrics showed no significant inter-group differences (**Figure 4A**; F denotes PD-1 intervention group). However, beta-diversity analysis revealed significant shifts in overall microbial community composition (R² = 0.162, p = 0.022) (**Figure 4B**), demonstrating that GR-4 plus PD-1 combinatorial therapy alters gut microbiota profiles in mice. Venn-diagram analysis illustrated OTU distribution overlap across experimental groups (**Figure 4C**). Genus-level gut-microbiota profiling (**Figure 4D**) showed that GR-4 intervention increased relative abundance of the probiotic genus *Ligilactobacillus* (control vs GR-4: 2.81% vs. 2.86%; PD-1 vs GR-4/PD-1: 1.41% vs. 4.04%). Relative abundance of *Bacteroides* also trended upward following combined GR-4-PD-1 treatment (control vs GR-4/PD-1: 2.51% vs. 4.63%). *Lactobacillus* abundance increased upon GR-4 and/or PD-1 exposure (control vs GR-4 vs PD-1 vs GR-4/PD-1: 2.68% vs. 5.11% vs. 4.90% vs. 3.12%). These results confirm that GR-4 beneficially modulates gut microbiota composition. At the phylum level, GR-4 intervention significantly enriched the probiotic phylum *Bacteroidota* relative to controls (p = 0.0268) (**Figure 4E**). At the class level, GR-4 treatment increased *Bacteroidia* abundance (p = 0.0255) and reduced the abundance of harmful *Actinobacteria* (p = 0.0441) (**Figure 4F**). At genus level, the GR-4/PD-1 group displayed significantly higher *Ligilactobacillus* proportions compared with the PD-1-only group (p = 0.0176) (**Figure 4G**). At species level, GR-4 supplementation significantly enriched short-chain-fatty-acid-producing commensal species *Agathobaculum desmolans*, *Lachnoanaerobaculum umeaense*, and *Lactobacillus fermentum* (p < 0.01) (**Figure 4H**). Collectively, both GR-4 monotherapy and combined GR-4-PD-1 treatment effectively restructure gut microbiota and enrich beneficial commensal taxa.

**Figure 4:**
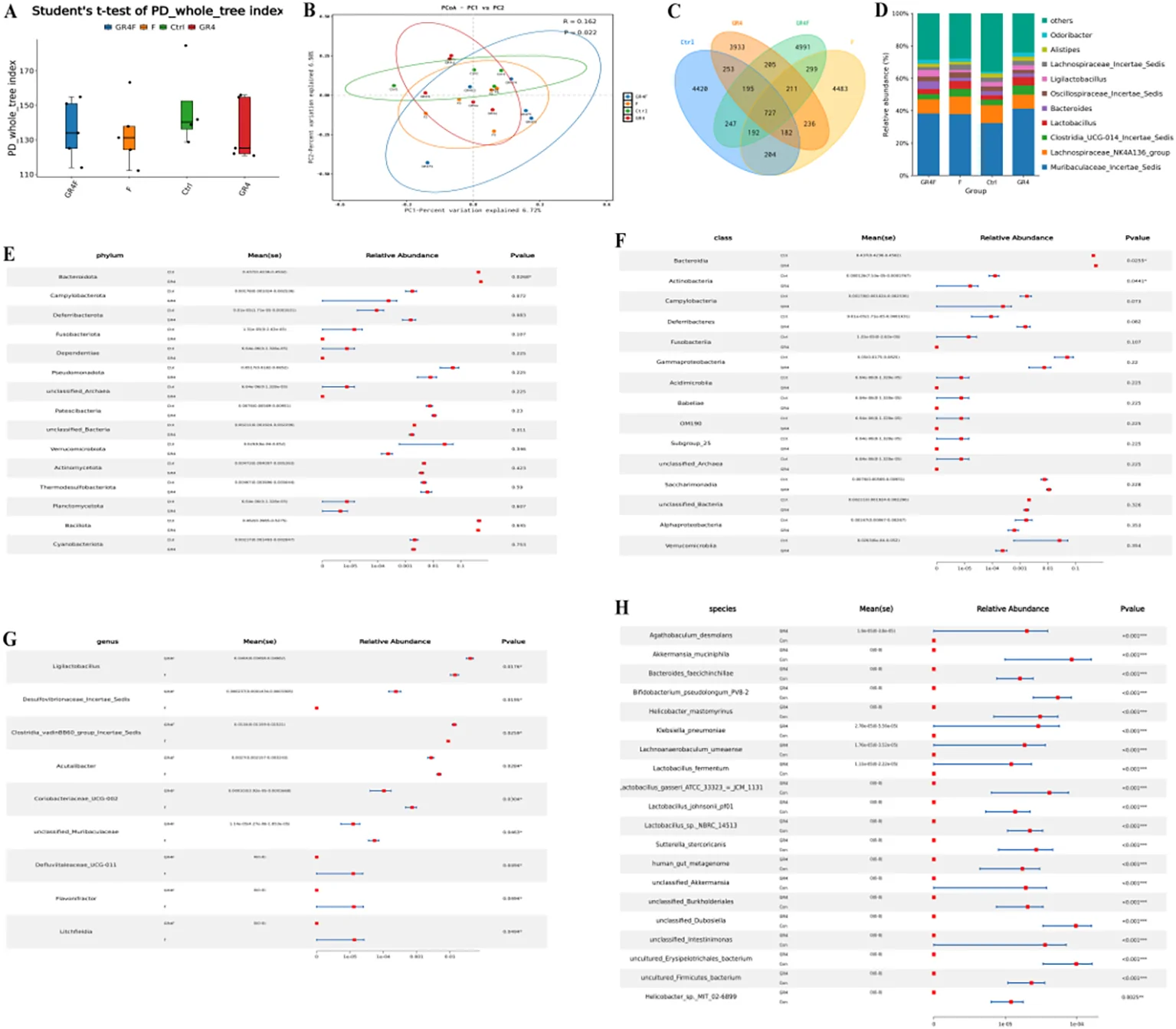
Effect of GR-4 on the gut microbial community. **(A)** Alpha diversity analysis of microbial composition. **(B)** Beta diversity analysis of microbial composition. **(C)** Venn diagram showing the number of shared and differential bacterial groups among groups. **(D)** Bar chart of the distribution of bacterial groups at the genus level in each group. **(E)** Composition differences of bacterial groups at the class level in each group. **(F)** Composition differences of bacterial groups at the phylum level in each group. **(G)** Composition differences of bacterial groups at the genus level in each group. **(H)** Composition differences of bacterial groups at the species level in each group.

### 3.5 GR-4 improves the gut microbiota and affects host immune function by regulating gut metabolites

Having established that GR-4 reshapes gut-microbiota composition, we next performed faecal metabolomics to decipher how microbial changes regulate host immune function (**Figure 5**). Distinct metabolite profiles separated the GR-4/PD-1 combination group from the PD-1-monotherapy group (**Figure 5A**), and separated the GR-4-monotherapy group from the control group (**Figure 5B**). Heatmap visualizations recapitulated these inter-group metabolic differences (**Figures 5C, D**). Volcano plots showed 51 significantly up-regulated and 35 significantly down-regulated metabolites in the GR-4 group versus controls (**Figure 5E**). Compared with PD-1 monotherapy, the GR-4/PD-1 combination group exhibited 146 significantly up-regulated and 62 significantly down-regulated metabolites (**Figure 5F**). Among top-ranked differential metabolites, ethoxyacetic acid (a short-chain-fatty-acid-related metabolite) was increased 1.52-fold in GR-4-treated mice relative to controls (**Figure 5G**). The antioxidant metabolite 2,5-dihydroxybenzoic acid was up-regulated 2.11-fold in the GR-4/PD-1 group relative to PD-1 monotherapy (**Figure 5H**). KEGG enrichment for differential metabolites revealed that metabolites altered by GR-4 monotherapy mapped to arachidonic-acid metabolism, butanoate metabolism, and multiple amino-acid metabolic pathways (**Figure 5I**). Differential metabolites from the GR-4/PD-1 versus PD-1 comparison were also enriched in arachidonic-acid and butanoate metabolism pathways alongside diverse amino-acid biosynthetic and catabolic pathways (**Figure 5J**). These findings indicate that GR-4-mediated gut-microbiota remodelling perturbs intestinal butanoate (short-chain-fatty-acid) metabolism as well as arachidonic-acid metabolism, a pathway closely linked to host inflammatory and immune responses.

**Figure 5:**
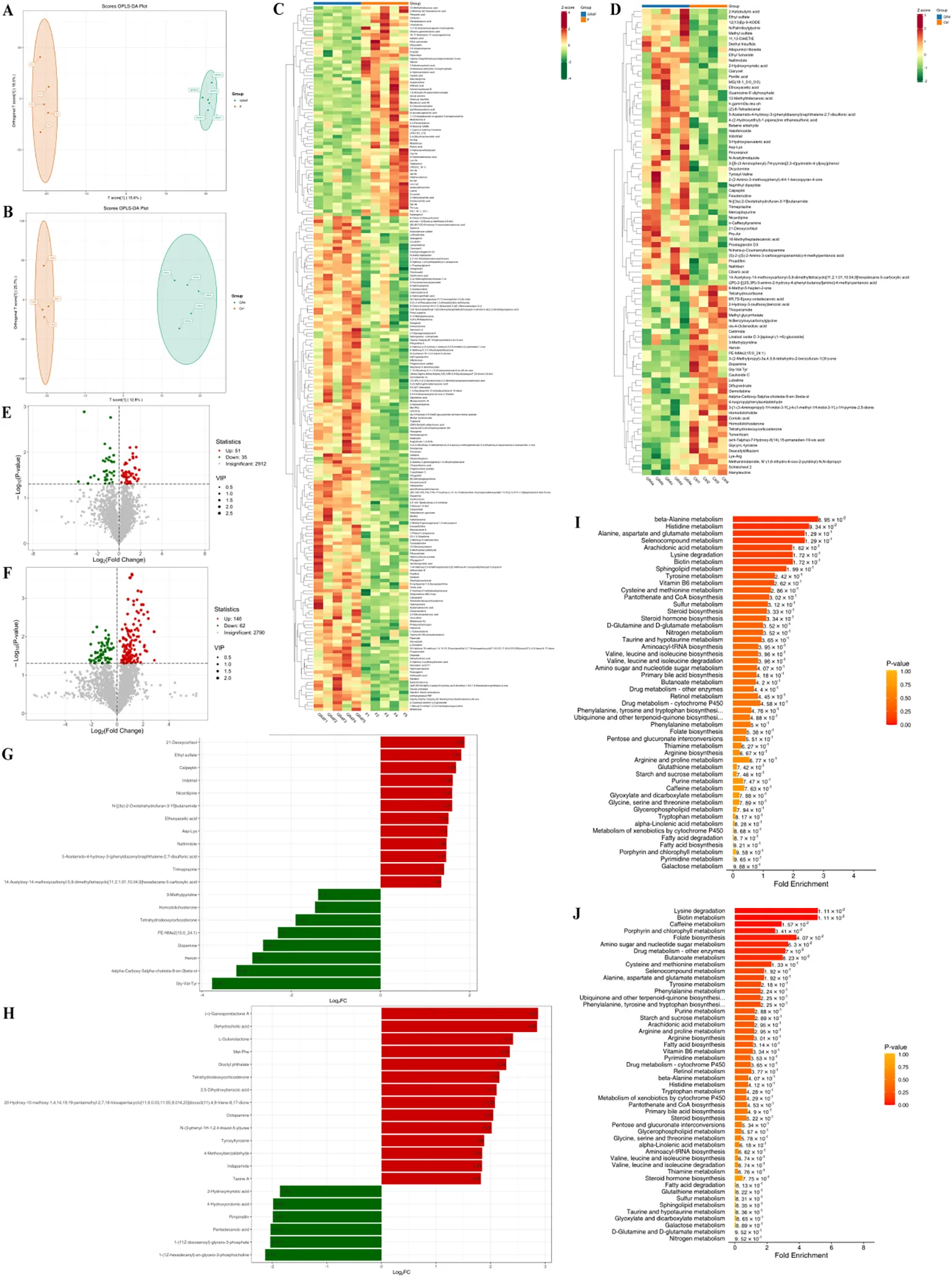
Effect of GR-4 on gut metabolites **(A)** OPLS-DA plot between the combined intervention group and the PD-1 alone intervention group. **(B)** OPLS-DA plot between the control group and the GR-4 alone group. **(C)** Heatmap between the combined intervention group and the PD-1 alone intervention group. **(D)** Heatmap between the control group and the GR-4 alone group. **(E)** Volcano plot between the combined intervention group and the PD-1 alone intervention group. **(F)** Volcano plot between the control group and the GR-4 alone group. **(G)** Main differential metabolites between the combined intervention group and the PD-1 alone intervention group. **(H)** Main differential metabolites between the control group and the GR-4 alone group. **(I)** KEGG pathway enrichment results of main differential metabolites between the combined intervention group and the PD-1 alone intervention group. **(J)** KEGG pathway enrichment results of main differential metabolites between the control group and the GR-4 alone group.

### 3.6 GR-4 may modulate immune responses via targeting the PD-1 signaling pathway

Our 16S sequencing and faecal metabolomics analyses demonstrated that GR-4 combined with PD-1 blockade modulates host immunity through gut-microbiota and metabolite alterations. To further characterize direct molecular mechanisms, we performed transcriptome sequencing on *in-vitro*-cultured human BGC-823 gastric cancer cells treated with GR-4 and/or PD-1 intervention. GR-4 substantially rewired BGC-823 transcriptomic profiles; clear separation was observed among control (MRS vehicle), PD-1, GR-4, and GR-4/PD-1 groups (**Figure 6A**). Heatmaps highlighted pronounced transcriptomic differences between control versus GR-4 groups (**Figure 6B**) and PD-1 versus GR-4/PD-1 groups (**Figure 6C**). Volcano-plot visualization showed 109 significantly up-regulated and 78 significantly down-regulated genes in GR-4-treated cells relative to controls (**Figure 6D**). Comparing GR-4/PD-1 against PD-1-only conditions yielded 194 significantly up-regulated and 137 significantly down-regulated genes (**Figure 6E**). KEGG pathway enrichment showed that DEGs from control-versus-GR-4 comparisons were enriched for the “PD-L1 expression and PD-1 checkpoint pathway in cancer” (**Figure 6F**). DEGs comparing GR-4/PD-1 against PD-1 monotherapy were also enriched in this same checkpoint-signalling pathway (**Figure 6G**). Enrichment-score quantification revealed statistically significant enrichment for PD-1-checkpoint-pathway genes in control-versus-GR-4 contrasts (p = 0.01) (**Figure 6H**), whereas enrichment for this pathway in PD-1-versus-GR-4/PD-1 comparisons did not reach statistical significance (p = 0.14) (**Figure 6I**).

**Figure 6:**
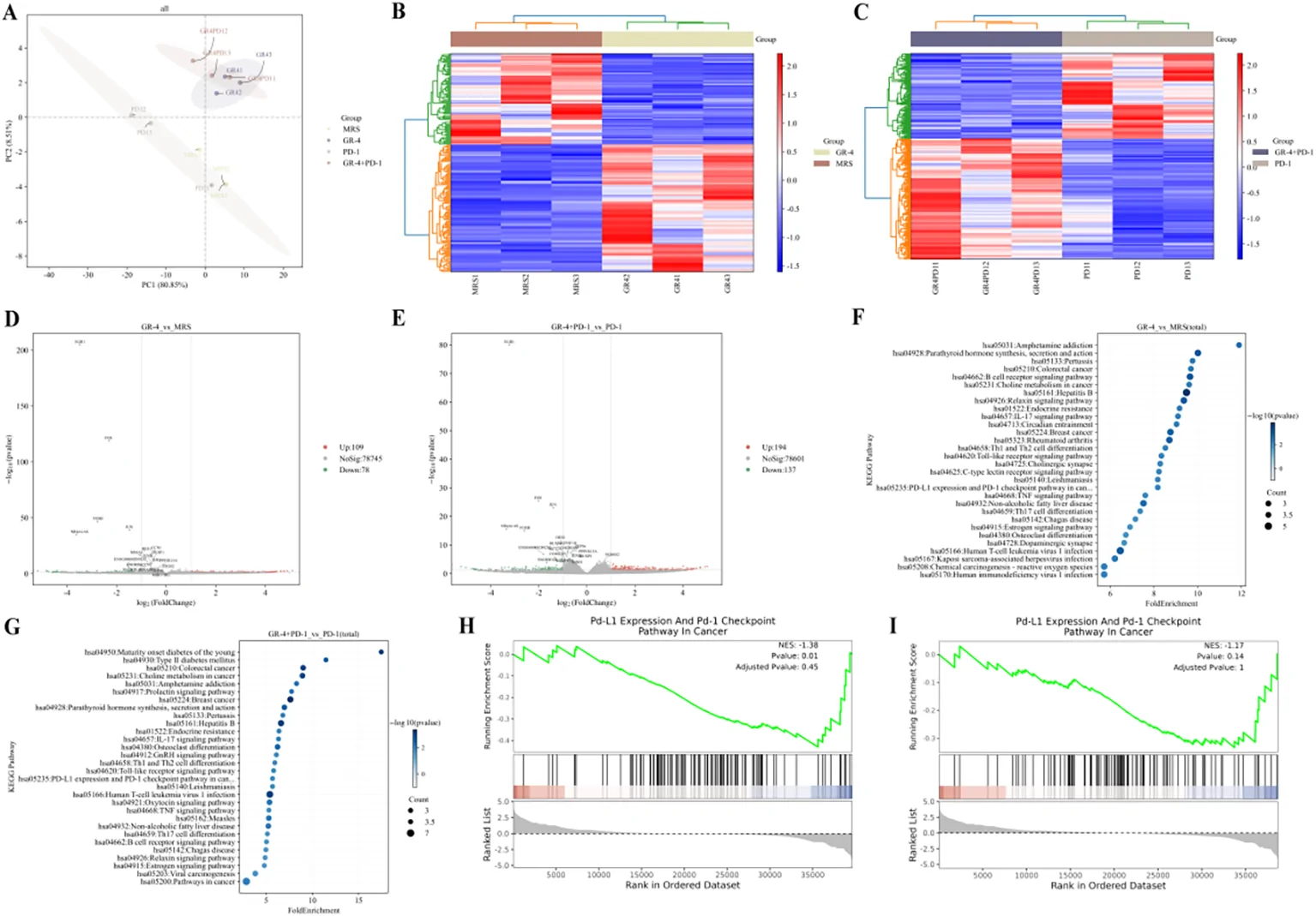
Effect of GR-4 on the gene expression profile of BGC-823. **(A)** PCA analysis results of the gene expression profile. **(B, C)** Heatmap analysis between different groups. **(D, E)** Volcano plot analysis between different groups. **(F, G)** KEGG enrichment analysis of intergroup differential genes. **(H, I)** Score of differential genes enriched in the PD-L1 expression and PD-1 checkpoint pathway in cancer.

## 4 Discussion

The gut microbiota constitutes a highly diverse, dynamically balanced microbial ecosystem that orchestrates host immune and metabolic signalling networks (20). Tumour development disrupts gut microbial homeostasis and reduces the abundance of beneficial commensal taxa (21); tumour-associated dysbiosis in turn drives multiple immune defects (22). Accordingly, restoring gut-microbiota homeostasis represents one promising strategy for improving cancer immunotherapy outcomes, and probiotic-based interventions have attracted growing attention.

Existing research has largely focused on indirect antitumor functions of probiotics mediated by gut-microbiota restoration and enhanced systemic immune responses. For example, selected probiotic strains improve intestinal barrier integrity and modulate host immune reactivity (23). Certain *Lactobacillus* strains exert anti-gastric-cancer activity by suppressing *Helicobacter pylori* colonization or modifying polyamine metabolism and apoptosis-related protein expression (e.g., regulating Bax/Bcl-2 ratios) in gastric epithelial cells (24, 25). In addition, microbiota profiling of gastric-cancer patient tissues has identified enriched *Lactobacillus* abundance, pointing to a potential link with the gastric tumour microenvironment. Nevertheless, most prior work only describes indirect tumour-suppressive effects or validates direct cytotoxicity in simplified tumour-cell monocultures lacking immune-cell components. Whether probiotics can directly sensitize gastric cancer to PD-1-based immune-checkpoint blockade — i.e., boost immune-system (especially T-cell-dependent) tumour recognition and killing — still lacks comprehensive *in-vivo* and *in-vitro* experimental evidence, and this scientific gap urgently requires investigation.

Jiangshui is a traditional fermented food indigenous to Northwest China, rich in probiotics, organic acids and bioactive compounds. It has documented physiological benefits including facilitating digestion, lowering serum cholesterol, and sustaining intestinal homoeostasis (11). Lactic acid bacteria serve as core functional microbes during Jiangshui fermentation: they produce lactic acid from carbohydrate substrates, inhibit pathogenic bacterial expansion, preserve mucosal-barrier integrity, and strengthen host immune defence (26). Furthermore, bacteriocins and other antimicrobial peptides secreted by lactic acid bacteria carry low resistance-inducing risk. Beyond anti-infective applications, recent work suggests their potential for restraining colorectal tumour progression (13). Despite confirmed safety for Jiangshui-derived lactic-acid-bacteria isolates, investigations into their key functional genes and downstream molecular mechanisms remain limited.

The current study demonstrates that *Lactobacillus plantarum* GR-4 synergizes with PD-1 immunotherapy to suppress tumour growth and mitigate immunotherapy-associated systemic toxicity *in vivo*. Multiple previous reports have highlighted the therapeutic potential of specific probiotic strains in oncology. For instance, microencapsulated *Lactobacillus plantarum* LAB12 reduces tumour burden in orthotopic colorectal-cancer mouse models via p53/caspase-3 up-regulation and COX-2/VEGF down-regulation (27). Exopolysaccharides derived from *Lactobacillus plantarum* also exhibit immunomodulatory activity, triggering secretion of pro-inflammatory cytokines IL-12 and TNF-α and supporting host antitumor immune responses (28). However, most prior studies centre on colorectal and other gastrointestinal malignancies; the effects of probiotics against gastric cancer and their combinatorial potential with immune-checkpoint inhibitors remain under-explored. Our *in-vivo* experiments show that GR-4 elevates circulating levels of antitumor-related cytokines such as IL-2 and IFN-γ, remodels intratumoral T-cell infiltration, and modulates T-cell CD69 activation markers, thereby reshaping the tumour immune microenvironment and generating synergistic anticancer effects together with PD-1 antibody. These observations align with existing clinical data linking intratumoral CD3^+^CD8^+^ T-cell density and spatial proximity to tumour cells with improved patient survival (29). High CD8^+^ T-cell infiltration is well-established as an independent biomarker predicting favourable immunotherapy responses and clinical outcomes across multiple solid-tumour types including non-small-cell lung cancer and colorectal cancer (30–32). Nevertheless, this study has notable limitations. We mainly evaluated T cell infiltration in subcutaneous tumors. In authentic gastric cancer, immune cells including T cells, B cells, macrophages, dendritic cells and NK cells, together with cytokines, jointly constitute the gastric cancer immune microenvironment, in which T cells represent only one component. It remains unclear how much anti-tumor contribution T cells make within the whole immune microenvironment. Moreover, the impact of GR-4 on the distribution of other immune cells was not investigated in the present work. More importantly, T cell exhaustion is one of the most critical challenges in current tumor immunotherapy, and the effect of GR-4-assisted immunotherapy on T cell exhaustion was not addressed in our study.

Probiotics are key modulators of intestinal immunity and can indirectly remodel the tumour microenvironment by tuning host systemic immunity. We show here that *Lactobacillus plantarum* GR-4, whether administered alone or combined with PD-1 blockade, remodels gut-microbiota composition and enriches commensal genera *Ligilactobacillus* and *Bacteroides*. Published work confirms that selected *Lactobacillus* strains enhance cytotoxic T-cell activation and intratumoral accumulation to improve immune-checkpoint-inhibitor efficacy (33). *Ligilactobacillus salivarius* was reported to potentiate PD-1 blockade by secreting bacterial extracellular vesicles that alter the tumour microenvironment, conferring clinical benefits for gastric-cancer patients (34). In addition, *Ligilactobacillus* metabolites such as indole-3-lactic acid maintain intestinal immune balance and mucosal immunity and ameliorate inflammatory ageing in pre-clinical mouse models (35). Intestinal commensal microbes commonly shape host immune homeostasis via short-chain-fatty-acid metabolites (36, 37), consistent with previous findings from our research group (38). Our metabolomic profiling further identified GR-4-associated increases in ethoxyacetic acid and 2,5-dihydroxybenzoic acid. Pathway enrichment linked GR-4 intervention to butanoate (short-chain-fatty-acid) metabolism and arachidonic-acid metabolism, a central pathway governing inflammation and immune function (39). Finally, Relative to the Ctrl group, GR-4 alone significantly altered the response of the PD-L1/PD-1 checkpoint pathway. In contrast, GR-4 did not exert a significant effect on this pathway in the presence of PD-1 inhibitor (PD-1 inhibitor group versus GR-4/PD-1 inhibitor group). This may be attributed to the fact that PD-1 inhibitor treatment strongly modulates the PD-L1/PD-1 checkpoint pathway, and the addition of GR-4 therefore does not induce further significant changes in pathway response.

In this study, we established an *in vivo* syngeneic gastric cancer model to preliminarily explore the adjuvant effect of GR-4 on gastric cancer immunotherapy. Our preliminary findings show that under combined intervention with GR-4 and PD-1 inhibitor, GR-4 may affect intestinal metabolites via gut microbiota and exert adjuvant anti-tumor effects by regulating the distribution of tumor-infiltrating T cells through the PD-1 signaling pathway. Integrating gut-microbiota profiling, metabolomics and transcriptomics, we demonstrate that GR-4 enriches gut commensals *Ligilactobacillus* and *Bacteroides*, modulates intestinal metabolites including ethoxyacetic acid, and interferes with the PD-L1-PD-1 cancer-checkpoint signalling axis. These events collectively alter intratumoral T-cell infiltration and produce synergistic anticancer activity with PD-1 blockade. Nevertheless, the present work relies primarily on high-throughput screening to generate mechanistic hypotheses, without targeted mechanistic validation. Follow-up work will perform dedicated *in-vitro* and *in-vivo* assays to dissect precise molecular cascades. Lactic-acid bacteria can regulate host immunity via surface peptidoglycans, teichoic acids, or secreted bioactive metabolites including short-chain fatty acids (40). Future studies should systematically identify GR-4-derived effector molecules driving antitumor immunity, for example by screening gene-knockout or overexpression mutant strains. Once key functional molecules are pinpointed, gene-knockout animal models or conditional knockout systems can validate T-cell-activating and tumour-microenvironment-remodelling pathways both *in vitro* and *in vivo*, establishing mechanistic insights into GR-4-mediated immune adjuvant activity. Furthermore, our *in-vivo* experiments employed subcutaneous syngeneic tumor models. The MFC cell line was derived from 615 mice (41). Thus, MFC cells were selected because they originate from 615 mice, which eliminates xenogeneic immune rejection in animal experiments. Meanwhile, when using an orthotopic gastric cancer model with daily oral administration of GR-4, the direct effect of GR-4 on gastric cancer cells cannot be ignored. Therefore, we established a subcutaneous syngeneic tumor models. Notably, the subcutaneous xenograft model used in the present study cannot faithfully recapitulate the native gastric tumor microenvironment, such as the unique acidic condition and gastric microbiota. These gastric-specific features may affect tumor progression, drug response and immune regulation. We had considered this limitation at the initial stage of project design. As the first report describing the adjuvant role of the probiotic GR-4 in tumor immunotherapy, this study aimed to answer two primary research questions: first, whether GR-4 exerts an effect on gastric cancer immunotherapy; second, to preliminarily explore the potential mechanism by which GR-4 assists gastric cancer immunotherapy. To address the first question, we adopted the simplest and most straightforward subcutaneous syngeneic tumor model for validation. For the second question, we integrated multi-omics analysis based on this subcutaneous syngeneic gastric cancer model to provide a panoramic overview of the preliminary mechanisms underlying GR-4-assisted gastric cancer immunotherapy. In this context, detailed issues such as the influence of GR-4 on T cell function and T cell exhaustion were not the primary focus of this manuscript.

In future follow-up investigations, we will establish an orthotopic gastric cancer model to better recapitulate the physiological conditions and tumor microenvironment of gastric cancer. We intend to elaborate the effects of GR-4 on the infiltration of multiple immune cells, as well as T cell function and T cell exhaustion during immunotherapy. We will also clarify the specific molecular mechanisms by which GR-4 assists gastric cancer immunotherapy through single-component intervention, metabolite target screening and other approaches.Gastric cancer frequently metastasizes to the liver, and the hepatic metastatic niche is heavily immunosuppressive and poses major challenges for combinatorial immunotherapy (42). Reproducing our key observations in larger-sample liver-metastasis or orthotopic gastric-cancer models will improve translational relevance. Patient-derived organoid systems co-cultured with autologous immune cells can also be deployed to evaluate personalized responses to GR-4 plus PD-1 inhibitor treatment in humanized experimental platforms, supporting precision stratification of potential beneficiary patient populations.

In summary, this study proposes and systematically characterizes a novel therapeutic strategy for gastric cancer: combining the traditional-food-derived probiotic *L. plantarum* GR-4 with PD-1 immune-checkpoint inhibitors. Conventional gastric-cancer therapies carry substantial toxicities, and although immunotherapy has delivered clinical breakthroughs, important limitations persist. Our work innovatively combines immunomodulatory probiotic resources originating from traditional Chinese fermented Jiangshui with modern tumour immunotherapy (PD-1 inhibitors). This creates new opportunities to overcome immunotherapy-resistance bottlenecks using safe, natural probiotic agents with cultural heritage, advancing translational research from basic mechanistic discovery toward clinical application. In the future, functional food products such as yoghurt or beverages enriched with *L. plantarum* GR-4 could be developed as oral dietary adjuvants for gastric-cancer patients receiving immunotherapy, facilitating convenient administration and improving therapeutic outcomes. This food-therapy combinatorial paradigm is conceptually innovative and bears broad prospects for gastric-cancer comprehensive management. Beyond translational applicability, this work also explores the molecular mechanisms whereby GR-4 remodels the tumour immune microenvironment and generates synergy with PD-1 inhibitors, conferring substantial scientific value and clinical-translational significance.

## Data Availability

The datasets presented in this study can be found in online repositories. The names of the repository/repositories and accession number(s) can be found in the article/supplementary material.
